# Acute Renal Infarction Heralds New-Onset Paroxysmal Atrial Fibrillation

**DOI:** 10.7759/cureus.21554

**Published:** 2022-01-24

**Authors:** Sindhura M Kolachana, Adrien Janvier

**Affiliations:** 1 Medicine, Georgetown University School of Medicine, Washington, DC, USA; 2 Medicine, MedStar Franklin Square Medical Center, Baltimore, USA

**Keywords:** hematuria, abdominal pain, anticoagulation, renal infarction, atrial fibrillation

## Abstract

Acute renal infarcts may be asymptomatic or occur with flank pain, nausea, vomiting, or hematuria. Given the non-specific symptomatology, many acute renal infarcts are misdiagnosed or not diagnosed at all. Most are diagnosed with contrast-enhanced computed tomography. A high index of suspicion should be maintained, especially for patients with cardiovascular risk factors. A negative workup for the etiology of a renal infarction should prompt cardiac monitoring for paroxysmal atrial fibrillation because this is the primary etiology in up to one-third of cases. Treatment of atrial fibrillation reduces the risk of recurrent renal infarction as well as stroke. Early diagnosis of acute renal infarction in a select group of patients may allow for endovascular intervention to re-establish vascular patency. Here, we review the case of a 43-year-old man with no significant medical history who presented with flank pain in the setting of an acute renal infarct.

## Introduction

An acute renal infarction occurs predominantly when a renal artery or any of its branches is blocked. The most common causes include thromboembolic phenomena, atherosclerosis, hypercoagulability, or traumatic injury [1]. Among thromboembolic phenomena, atrial fibrillation is the most common and well-documented etiology. Atrial fibrillation is more commonly associated with an increased risk of stroke; however, there are several reports of acute renal infarction caused by atrial fibrillation. Renal infarction of any etiology can have devastating outcomes, especially in individuals with pre-existing chronic kidney disease. Unfortunately, the presentation of renal infarction is often non-specific. Although patients commonly present with acute flank or abdominal pain, they may also present with non-specific symptoms such as nausea, vomiting, fever, or new-onset hypertension [2]. As such, acute renal infarction may mimic other more common conditions such as pyelonephritis, nephrolithiasis, pancreatitis, or appendicitis [3]. Endovascular intervention and subsequent revascularization are beneficial when renal infarcts are identified and diagnosed early in the disease process; however, given the non-specific presentation, early diagnosis is uncommon [4]. Clinicians should consider the possibility of renal infarction when a combination of flank pain, hematuria, and elevated lactate dehydrogenase is noted. A definitive diagnosis of renal infarction is often made with a contrast-enhanced computed tomography (CT) scan or a CT angiogram [2]. Anticoagulation is generally recommended, although the duration is determined on an individual basis. Here, we present the case of a 43-year-old man with an otherwise unremarkable medical history who presented with clinical and imaging findings consistent with a renal arterial thrombus and renal infarction, which was ultimately identified to be in the setting of paroxysmal atrial fibrillation.

## Case presentation

**Figure 1:**
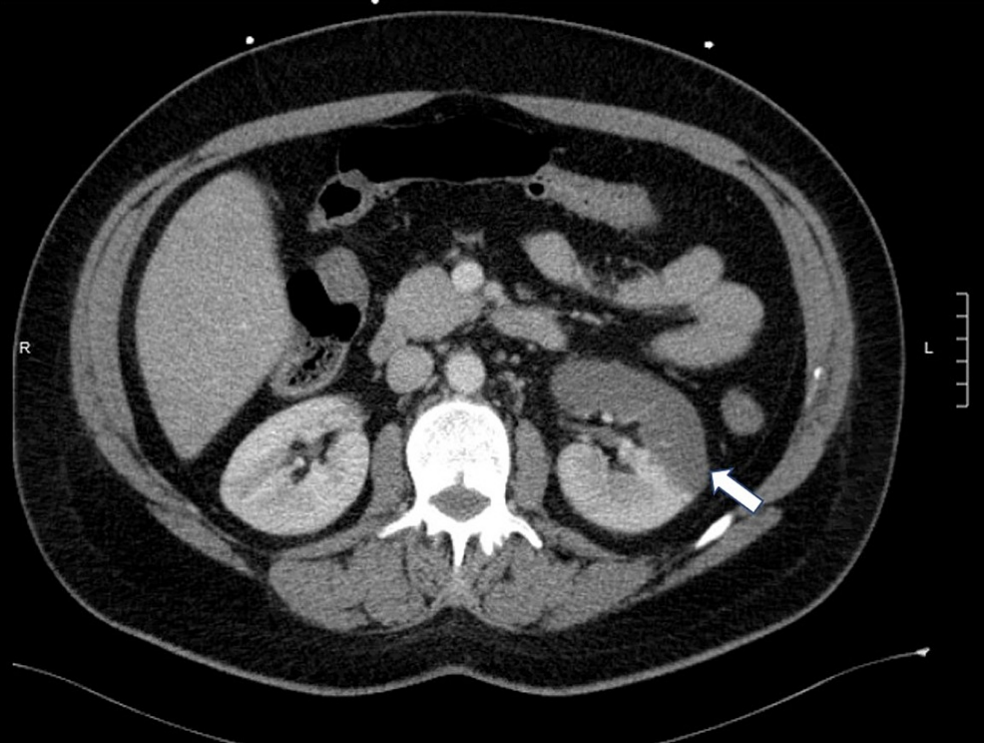
Axial computed tomography scan of the abdomen with contrast showing reduced enhancement of the anterior and caudal portion of the left kidney (white arrow) consistent with infarction.

**Figure 2:**
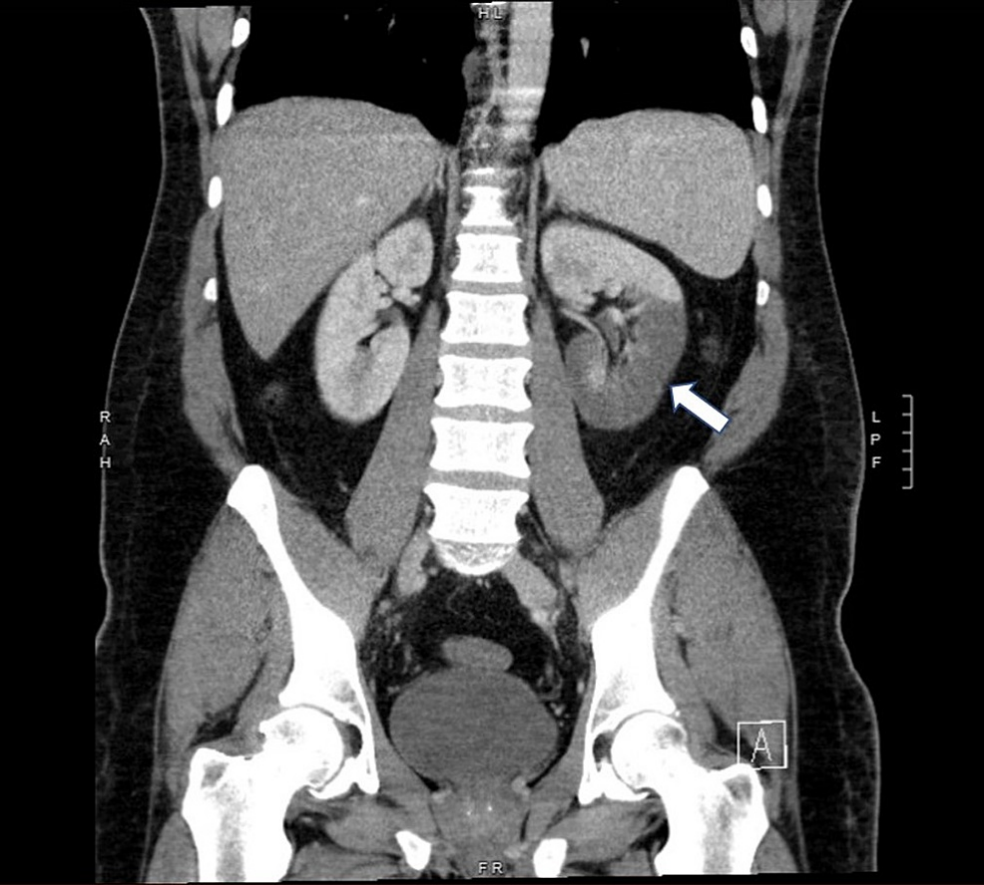
Coronal computed tomography scan of the abdomen with contrast showing reduced enhancement of the lower pole of the left kidney consistent with infarction.

**Figure 3:**
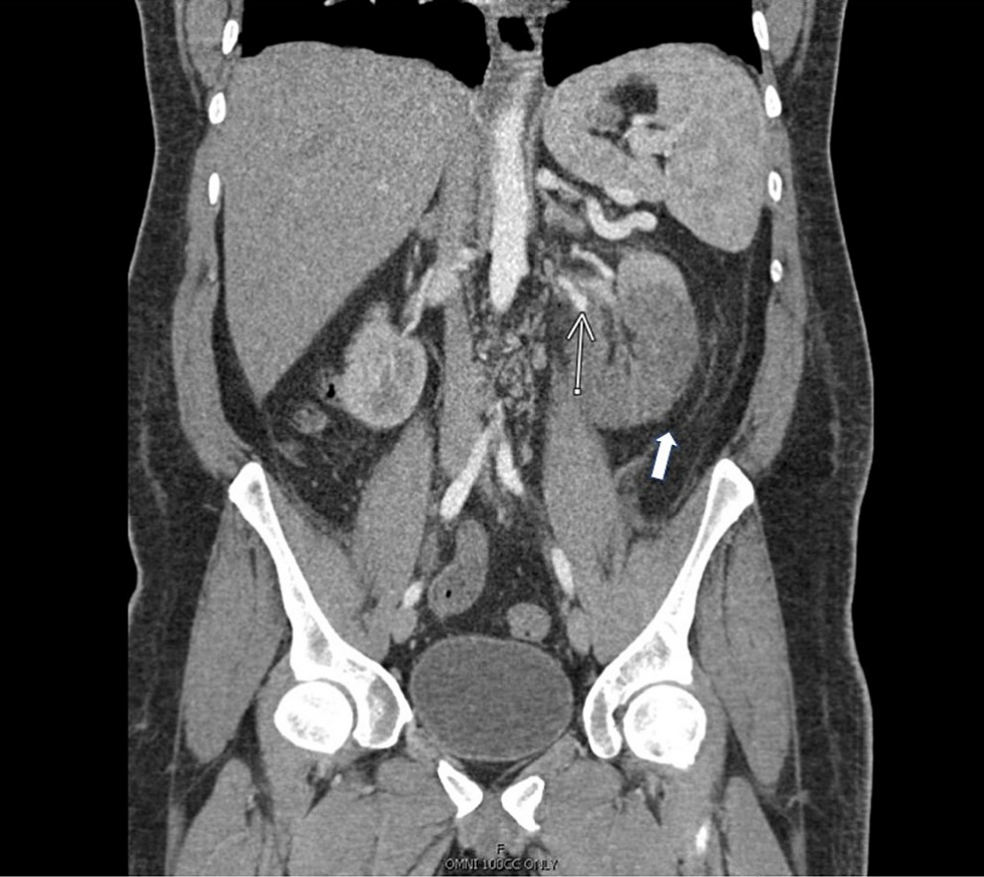
Computed tomography angiography of the abdomen. Note the reduced enhancement of the lower pole of the left kidney and a filling defect of the left renal artery (thin arrow). Also seen are perinephric fat stranding (thick arrow), patent left superior renal artery, and normal left upper renal pole enhancement.

A 43-year-old man with a history of asthma and a recently treated community-acquired pneumonia infection presented to the emergency room with a one-day history of worsening lower abdominal pain, nausea, and vomiting. He reported severe abdominal pain and had vomited five times before presenting to the hospital. On a review of the systems, he also reported a two-week history of intermittent sensation of chest pressure. On presentation, vitals were significant for a heart rate of 99 beats per minute, respiration rate of 18 breaths per minute, blood pressure of 202/83 mmHg, and oxygen saturation of 96%. On evaluation, he was in acute distress due to significant abdominal pain. Physical examination revealed tenderness to palpation to the left flank and all quadrants of the abdomen with rebound tenderness. Physical examination was also notable for a II/VI diastolic decrescendo murmur best heard at the upper right sternal border. Laboratory studies were remarkable for mild anemia (hemoglobin of 9.7 g/dL), mild leukocytosis (white blood cell count, 11,100/µL), slightly elevated lactic acid level (2.5 mmol/L), and negative troponin. CT scan of the abdomen and pelvis with contrast demonstrated a left renal infarct involving the lower half of the kidney (Figures 1, 2) associated with perinephric stranding (Figure 3). A filling defect in an inferior division of the anterior left renal artery was identified, while patency to the superior division was preserved (Figure 3).

Given that the imaging findings were consistent with a thrombosed left inferior renal artery and associated left lower pole kidney infarction, a heparin drip was initiated. He was placed on telemetry empirically, and we incidentally noted an episode of atrial fibrillation lasting approximately two hours (heart rate, 130s) with spontaneous conversion to sinus rhythm. High-sensitivity troponin increased to 1,922 ng/L consistent with a type 2 myocardial infarction secondary to atrial fibrillation with a rapid ventricular response. Transthoracic echocardiogram was significant for moderate aortic regurgitation with preserved ejection fraction (56%). A pharmacologic stress test revealed no inducible ischemia. Remarkably, his creatinine was 0.8 mg/dL on admission and remained unchanged by day four of hospitalization. He was stabilized on metoprolol and warfarin therapy and advised to follow up with cardiology.

## Discussion

Renal infarcts almost certainly occur more often than diagnosed or documented. A study by Hoxie and Coggin in 1940 found 205 cases of renal infarcts out of 14,411 general autopsies performed at the county hospital. In their population of autopsied patients, approximately 1.4% sustained renal infarcts [5]. Because only two of those infarcts were diagnosed prior to death, the rate of clinical detection was approximately 1%, suggesting that renal infarcts are likely underdiagnosed. Despite advances in technology, the non-specific signs and symptoms associated with renal infarction contribute to diagnostic uncertainty. Patients commonly present with flank or abdominal pain; however, these may be due to a myriad of other causes. Even lactate dehydrogenase (LDH), one of the most sensitive markers for renal infarct, suffers from a lack of specificity [1,6-9]. LDH is also elevated in conditions such as myocardial infarction, liver pathology, and hemolytic processes [6].

Many conditions predispose to renal infarction such as atrial fibrillation, atheroembolic disease, coagulopathy, trauma, renal artery dissection, malignancy, and even coronavirus disease 2019 [10,11]. A systematic review of published studies on renal infarcts by Pizzarossa and Mérola included 1,582 patients and found the following probable causes of renal infarction: cardiac or aortic embolism (45%), arterial injury (16%), coagulopathy (9%), idiopathic (18%), and other (5%). Seventy-five percent of the cardiac/embolic cases were due to atrial fibrillation making it the most common individual cause [12]. Another review by Oh et al. identified 438 cases of renal infarct, 55% of which were cardioembolic in origin, and 48% of which were secondary to atrial fibrillation [1]. Given these findings, it is not surprising that the management of most acute renal infarcts includes anticoagulation. Anticoagulation is recommended for at least six months; however, some patients do well with antiplatelet therapy alone [13]. Anticoagulation appears to be beneficial but is associated with a risk of bleeding [14].

Less common interventions such as the use of fibrinolytics, surgical thrombectomy, and endovascular procedures are also utilized at the time of diagnosis based on a patient’s clinical presentation and status. The benefits likely outweigh the risks for patients with bilateral renal artery occlusion or solitary kidney. Caution is warranted with surgical embolectomy because it is associated with significant morbidity, and studies have indicated mixed outcomes in patients [15,16]. A retrospective review of cases of bilateral and unilateral renal artery occlusion by Haas and Spirnak showed that surgical revascularization was successful in 56% of cases with bilateral occlusion (n = 16) and 26% of cases with unilateral occlusion (n = 34). Further, decreased renal function was identified in 67% of individuals who had previously undergone successful unilateral revascularization [15]. The role of endovascular intervention in the management of acute renal infarction secondary to arterial thromboembolism is promising but is in the early stages of development. There are multiple case reports of successful re-establishment of renal artery patency with endovascular intervention [17]. Specifically, multiple case reports have utilized percutaneous rheolytic thrombectomy to successfully relieve an intravascular renal arterial thrombus due to atrial fibrillation [18,19]. It is generally thought that the greatest likelihood of benefit from revascularization therapy occurs within 24 hours of the onset of symptoms; however, patient characteristics are undoubtedly important. In one study, renal transplant patients did not fare as well after endovascular intervention despite less than 24 hours having elapsed from the onset of symptoms. Conversely, other patients demonstrated benefit from the intervention as far as 96 hours from the onset of symptoms [4]. Acute renal infarction carries the risk of reduced renal function or the need for hemodialysis. In one case series including 94 patients with acute renal infarction treated medically and/or surgically, seven patients required hemodialysis [20]. The risk of progression to end-stage renal disease is likely to be higher in patients who are undiagnosed or diagnosed late in the course of illness.

## Conclusions

It is important to have a low threshold of suspicion for acute renal infarction when a patient presents with flank pain, nausea, vomiting, and hematuria. Patients diagnosed early may be candidates for endovascular intervention. Given that atrial fibrillation is a common cause of acute renal infarction, it may make sense to screen for it in patients with no other obvious cause. Although pragmatic, studies supporting this recommendation are yet to be conducted. Lastly, most patients require anticoagulation for at least six months, but the duration of therapy should be individualized.
